# Development of a programmable magnetic agitation device to maintain colloidal suspension of cells during microfluidic syringe pump perfusion

**DOI:** 10.1371/journal.pone.0282563

**Published:** 2023-03-08

**Authors:** Tommy Puttrich, Steven O’Donnell, Sing-Wan Wong, Miiri Kotche, Anthony E. Felder, Jae-Won Shin

**Affiliations:** 1 The Richard and Loan Hill Department of Biomedical Engineering, University of Illinois at Chicago, Chicago, Illinois, United States of America; 2 Department of Pharmacology and Regenerative Medicine, University of Illinois at Chicago, Chicago, Illinois, United States of America; The Ohio State University, UNITED STATES

## Abstract

Droplet-based microfluidic devices have been used to achieve homogeneous cell encapsulation, but cells sediment in a solution, leading to heterogeneous products. In this technical note, we describe automated and programmable agitation device to maintain colloidal suspensions of cells. We demonstrate that the agitation device can be interfaced with a syringe pump for microfluidic applications. Agitation profiles of the device were predictable and corresponded to device settings. The device maintains the concentration of cells in an alginate solution over time without implicating cell viability. This device replaces manual agitation, and hence is suitable for applications that require slow perfusion for a longer period of time in a scalable manner.

## Introduction

Tissue engineering is a multidisciplinary area of research applying principles of biomaterial design and medicine to generate tissues and organs that better replicate their original functions and structures [1–5]. One technique used in tissue regeneration is the microscale encapsulation of single cells in a hydrogel for precision niche modeling and delivery via the use of droplet-based microfluidic devices [6–9]. To encapsulate cells, a colloid must be prepared consisting of the living cells and the aqueous phase of the material the cells will be encapsulated within. This colloid must be slowly perfused through a microfluidic device to create small capsules of cells in a monodisperse manner [10]. However, due to the high density of the cells, they sediment from the colloid over time.

Current methods to resuspend cells involve manual agitation using a small stir bar placed within the colloid, which is then manipulated externally using a neodymium magnet. Although effective at preventing premature cell aggregation, the task is manual, time-intensive, and may produce inconsistent homogeneity. To address this shortcoming, different types of automated stirring devices have been developed. In dispersive liquid–liquid microextraction applications, a rotating magnetic stir bar is used to induce a vortex in the syringe solution [11, 12], although this approach has not been demonstrated with live cell culturing. Lack of translation of the magnetic stir bar longitudinally along the syringe is an additional shortcoming. The commercially available Cetoni Nemix^TM^ system agitates a syringe solution by both translating and rotating a magnetic stir bar along the syringe axis [13]. However, this system is proprietary and integrated, which precludes use with additional third-party equipment and software. Another commercially available solution from GPD Global utilizes a screw drive to agitate a syringe solution [14]. This technique is intended for industrial manufacturing purposes with higher volumes and has also not been demonstrated with live cell culturing applications. Here, we design, fabricate, and evaluate an automated agitation device to maintain colloidal suspension of cells during syringe pump perfusion. This device is low-cost, modular, and adjustable to accommodate existing syringe pumps.

## Materials and methods

### Design of the programmable agitation device

**Fig 1:**
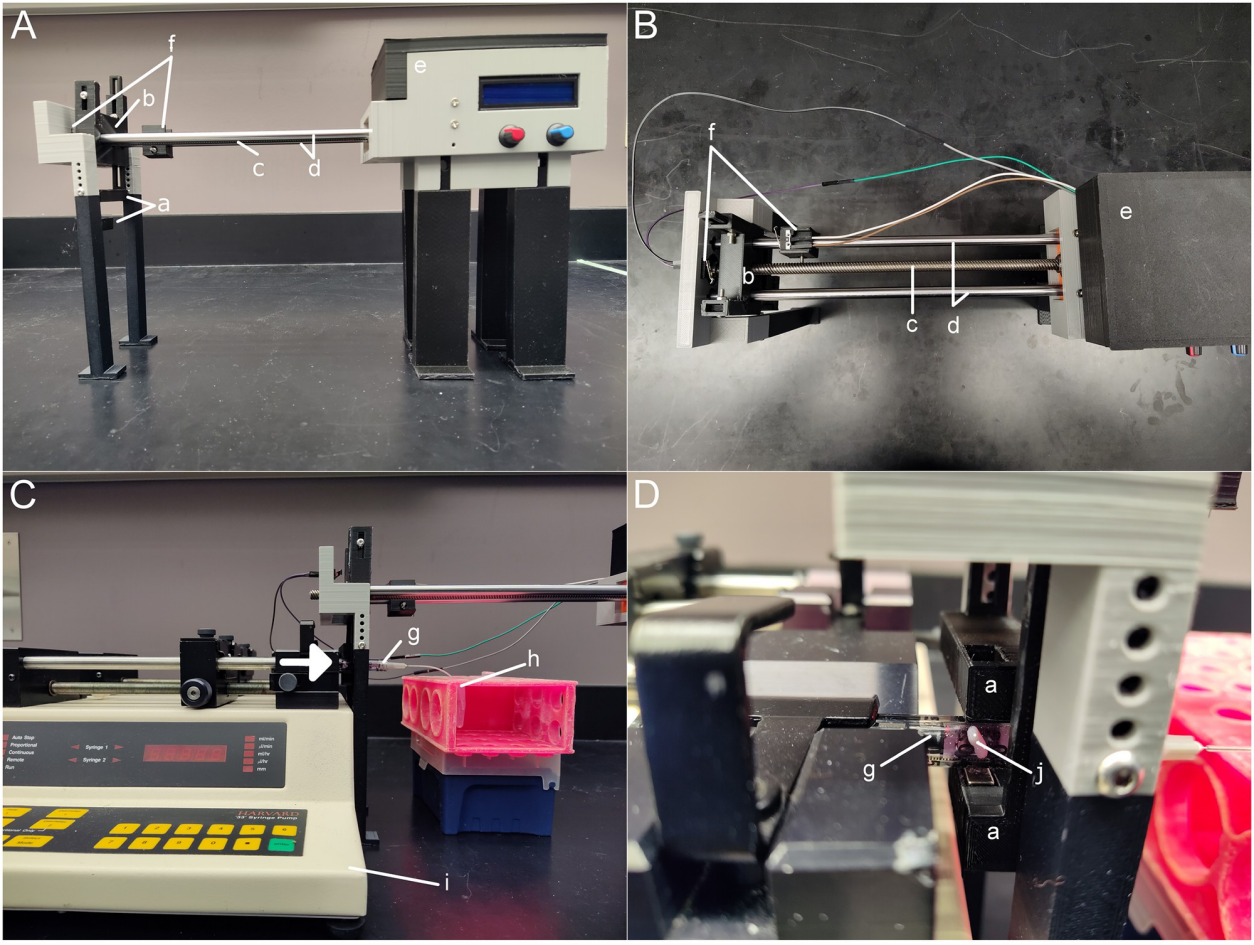
The automated agitation device to maintain colloidal suspension during syringe pump perfusion. Front (A) and top (B) views without a syringe pump. The device alongside a syringe pump from the front (C) with an arrow indicating perfusion direction and a closeup, transverse view of the syringe (D). Labeled components include the hooked attachments with mounted neodymium magnets (a), the lead screw assembly (b), the lead screw (c), guide rods (d), the controller housing (e) containing the DC motor, joint coupler, microcontroller, motor controller, potentiometers, push buttons, and liquid crystal display, the end-stop limit switches (f), a syringe (g), a collection Eppendorf tube (h), a syringe pump (i), and the magnetic stir bar (j).

The programmable agitation device was designed in Solidworks (version 2021, Dassault Systèmes) and 3D printed in polylactic acid using a fused deposition modeling printer (Ender 3 Pro, Creality). The device is modular and adjustable to accommodate horizontally-oriented syringe pumps commonly used in scientific research labs. As shown in Fig 1, the device is elevated using telescoping legs to accommodate a syringe pump (Harvard 33, Harvard Apparatus). The operational principle of the device is to linearly translate an external magnet along the length of a syringe barrel, which in turn translates a micro stir bar placed in the syringe. Motion of the micro stir bar agitates the fluid within the syringe and maintains suspension.

Neodymium magnets (N35, 10x5x3 mm) external to the syringe barrel are mounted to the lead screw assembly (Fig 1, component b) via independent vertically-adjustable hooked attachments (Fig 1, component a). The hooked attachments and external magnets are oriented to straddle the syringe barrel (Fig 1, component g). The external magnets define longitudinal positioning of the magnetic stir bar (Fig 1, component j, 2x5 mm, No. 58948–377, VWR) within the syringe. Linear translation of the lead screw assembly is provided via rotation of the lead screw (Fig 1, component c, 400 mm length, 8 mm diameter, 2 mm pitch, Uxcell). Rotation of the lead screw is provided by a brushed motor (200 RPM 12 VDC, Uxcell) with a flexible coupler joint located within a controller housing (Fig 1, component e). Rotation of the DC motor is controlled by a programmable microcontroller (Arduino Uno) and DC motor controller (DROK L298). A customized control software was written for the microcontroller using the Arduino IDE. The rotational speed of the lead screw and resulting translational speed of the lead screw assembly is controlled with an 8-bit PWM signal from the microcontroller to the motor controller. Two potentiometers and three button switches on the controller housing are used to define linear speed, period of translation, and advance menu options during setup. A 16x2 LCD screen mounted to the controller housing is used to visually indicate settings to the user. The length of translation of the lead screw assembly, and thus the external magnets, is defined using two end-stop limit switches (Fig 1, component f) which are affixed to the guide rods (Fig 1, component d) by a customized mount. During one complete translation cycle, the lead screw assembly translates forwards and then backwards between the distance defined by the end-stop limit switches. As the external magnets translate, the magnetic stir bar placed in the syringe also translates to agitate the solution. During operation, the frequency of translation, number of translation cycles, and total time of translation are displayed on the Arduino serial monitor and LCD screen. To accommodate the decreased throw distance resulting from the real-time depression of the plunger via the syringe pump, the software incorporates a function which decreases translation distance over time according to the rate of plunger movement. Supplemental video (S1 Video) demonstrates a closeup view of hooked attachments with mounted neodymium magnets straddling the syringe and magnetic stir bar. The device executes multiple translation cycles at an average speed of ∼33 mm/s, agitating the HBSS media within the syringe.

### Verification of translation velocity

One potentiometer coordinates an 8-bit PWM signal to the DC motor controller, with an effective maximum and minimum translational velocity of the lead screw assembly being 42.86 mm/s (255 PWM signal) and 14.77 mm/s (50 PWM signal—the minimum signal to power the DC motor). To ensure the velocity of the external magnets corresponds to the stir bar magnet velocity, an evaluation was performed. Stir bars were placed into separate syringes containing Hank’s Balanced Salt Solution (HBSS, Thermo Fisher Scientific) and alginate (LF200, FMC Polymer) media, representing different viscosities and resistances to translation of the stir bar magnet. Syringes were mounted to the Harvard Apparatus 33500TM syringe pump and the agitation system was activated at varying speeds. The stir bar motion within the syringe barrel was captured by video recording for each agitation condition. Captured videos were analyzed using a customized image analysis algorithm written in MATLAB (version 2015, MathWorks) for semi-automated measurement of kinetic linear velocity. The algorithm first auto-calibrates the spatial resolution of a video using the known stir bar principal dimension (5 mm) and then tracks user-inputted mouse clicks as frames are manually advanced. Each mouse click corresponds to an X-Y location in the frame of a defining feature for the stir bar (e.g., its lower-right corner) that remains consistent across frames and translation. Mouse clicks are tracked across frames to measure a linear distance and calculate a velocity. Six linear motions were tracked for each condition/video, with an average and standard deviation of velocities being calculated. The velocities correspond to kinetic motion of the stir bar after static friction was overcome.

### Cell culture

Clonally derived D1 mouse mesenchymal stromal cells (MSCs; American Type Cell Culture, CRL-12424) were used since they were used by a number of studies on cell-material interactions in the context of tissue regeneration [15, 16]. Cells were cultured in complete high glucose Dulbecco’s Modified Eagle Media (DMEM; Thermo Fisher Scientific) supplemented with 10% fetal bovine serum (FBS; S11550, Atlanta Biologicals), 100 units/mL penicillin and 100 *μ*g/mL streptomycin, and 2 mM GlutaMAX (Thermo Fisher Scientific). Cells were passaged when they reached 80% confluence in a 175 cm^2^ flask by detaching with trypsin-EDTA (Thermo Fisher Scientific). Passage numbers less than 15 were used for this study.

### Verification of agitation

To test the ability of the agitation device to maintain cell suspension, detached cells were washed twice with HBSS and resuspended in either HBSS or 1% w/v high molecular weight (∼240 kDa) alginate (LF200; FMC Polymer) solution in complete DMEM at 5 million cells/mL. 500 *μ*L of the cell suspension was loaded into a 1-mL syringe (4.78mm inner diameter; BD #309628). Syringes had a stir bar placed inside prior to loading of the cell suspension. The syringes were mounted to a Harvard Apparatus 33500TM syringe pump. The automated agitation device was assembled and mounted adjacent to the syringe pump. The syringe pump was set to perfuse 1 *μ*L/min and the dispensed liquid was collected using Eppendorf tubes. Colloidal suspension concentrations were measured over two hours at the following time points: 0, 15, 30, 60, 90, and 120 min. Eppendorf tubes were replaced at every time point to preserve solution measurements. Four experimental agitation conditions were evaluated: no agitation, manual agitation, low-rate continuous agitation (velocity of ∼14 mm/s), and high-rate continuous agitation (∼42 mm/s). The manual agitation condition was applied by hand as previously described [6–9], and served as a positive control. Cell density of the dispensed solutions was determined via counting using a hemocytometer.

### Gene expression analysis

Cells in suspension were collected at the 120-min time point after stirring and treated with 100 *μ*L of alginate lyase (3.4 mg/mL in HBSS; Sigma) for 30 min at 37°C. The samples were then washed with HBSS followed by 900g centrifugation. Supernatant was aspirated and the cell pellet was lysed in 500 *μ*L of Trizol (Thermo Fisher Scientific) for 10 min at room temperature. Trizol digested samples were stored at −80°C up to 2 weeks if not processed immediately. Chloroform (100 *μ*L) was added to each Trizol sample and shaken vigorously for phase separation. Samples were then centrifuged for 10 min at 14,686g at 4°C. After separation, the top layer which containing RNA was transferred into a new tube, and the RNA was precipitated with 250 *μ*L of 100% isopropanol and 250 *μ*L of 0.8 M sodium citrate plus 1.2 M sodium chloride in RNase-free water for at least 15 min at 4°C. Samples were then centrifuged at 14,686g for 10 min at 4°C. The supernatant was removed, and the precipitated RNA was washed with 75% v/v (12.8 M) ethanol in RNase-free water. The samples were briefly vortexed then centrifuged for 5 min at 5,287g at 4°C. After removing ethanol, purified RNA was resuspended in 15 *μ*L of RNase-free water. The concentration and quality of purified RNA was measured by NanoDrop spectrophotometer (Thermo Fisher Scientific). Complementary DNA was synthesized by reverse transcription using SuperScript-III reverse transcriptase (Thermo Fisher Scientific). 50 ng of complementary DNA was used for quantitative PCR (qPCR) analysis with Low ROX Forget-Me-Not EvaGreen Master Mix (Biotium). Each sample was analyzed in triplicate. qPCR was performed in the ViiA7 qPCR system (Applied Biosystems) with QuantStudio 7 software (v1.3). Relative gene expression was reported with the 2^-ΔC_t_^ method by normalizing the cycle threshold (C_t_) value of each target gene to the reference gene (*Gapdh*). Table 1 shows the list of primers used in this study.

**Table 1:** Primers used for gene expression.

Gene	Primer
*Acta2*	F: GTGAAGAGGAAGACAGCACAG
	R: GCCCATTCCAACCATTACTCC
*Ctgf*	F: CTCCACCCGAGTTACCAATG
	R: TGGCGATTTTAGGTGTCCG
*Piezo1*	F: TACTCCAGCAACTGCACTGA
	R: CCAGCAACAGAAGGATCTGC
*Gapdh*	F: CTTTGTCAAGCTCATTTCCTGG
	R: TCTTGCTCAGTGTCCTTGC

### Statistical analyses

Linear regression was used to evaluate the relation between external magnet and micro stir bars velocities. Two-way ANOVA was used to determine effects of time and agitation conditions on colloidal concentration. If a significant interaction was detected, simple main effects were evaluated using one-way ANOVA with Tukey post-hoc. The effects of agitation conditions on the standard deviation of colloidal concentrations and viability of cells at 120 minutes was determined by one-way ANOVA. All statistical analyses were performed using Prism (version 9.3.1; GraphPad Software) and SPSS (v 26; IBM). Statistical significance was accepted at p-value less than 0.05.

## Results

### Verification of stir bar velocity

**Fig 2:**
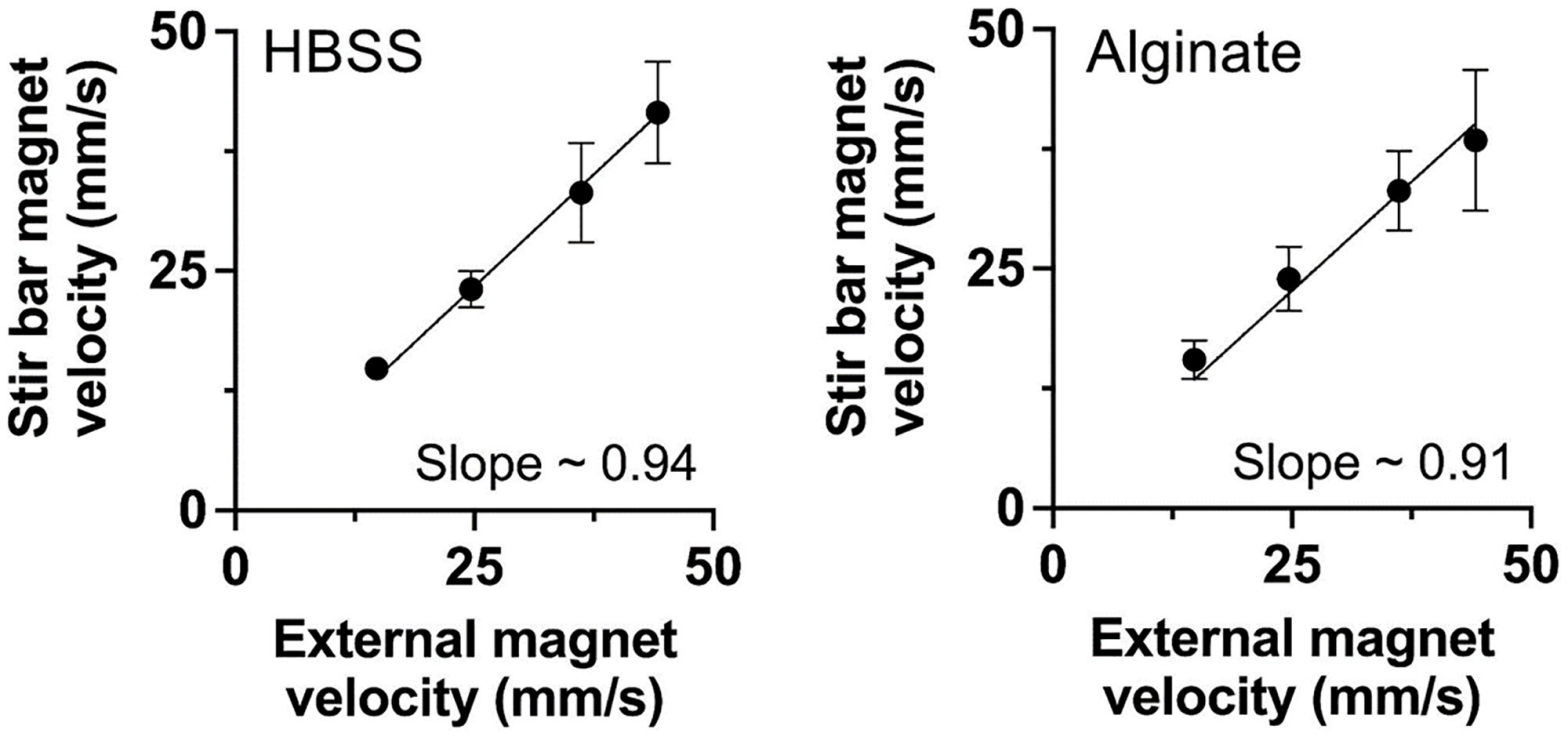
Stir bar velocity in a syringe can be externally controlled. The velocity of the stir bar in HBSS (left) or 1% w/v alginate (right) solution was measured with varied external magnet velocity. Best fit lines were plotted using linear regression with y-intercept = 0 (R^2^>0.96). All data are from n = 3 independent experiments. Error bars indicate standard deviation.

To test the ability of the agitation device to predictably translate the internal stir bar magnet, the relationship between velocity of the internal stir bar and that of the external magnets mounted to the lead screw assembly was determined (Table 2). Across all evaluated velocities, mean error was less than 14%, with an average of 6.35%. From the plots of stir bar magnet velocity vs. external magnet velocity (Fig 2), linear best fit lines for HBSS and alginate (1% w/v) media had slopes of 0.94 and 0.91, respectively. The velocity of the internal stir bar generally corresponds to that of the external magnets and can be calibrated for each medium.

**Table 2:** Translational velocities of both the external and stir bar magnets during agitation.

External magnet Velocity (mm/s)	Stir bar magnet velocity in HBSS media (mm/s)	Mean error in HBSS media (%)	Stir bar magnet velocity in alginate media (mm/s)	Mean error in alginate media (%)
14.77	14.83 ± 0.97	0.41	15.51 ± 2.03	5.03
24.68	23.11 ± 1.89	6.38	23.94 ± 3.37	3.01
36.18	33.16 ± 5.20	8.34	33.12 ± 4.16	8.46
44.20	41.54 ± 5.23	6.02	38.40 ± 7.36	13.11

### Automatic agitation maintains viable cells in suspension over time

**Fig 3:**
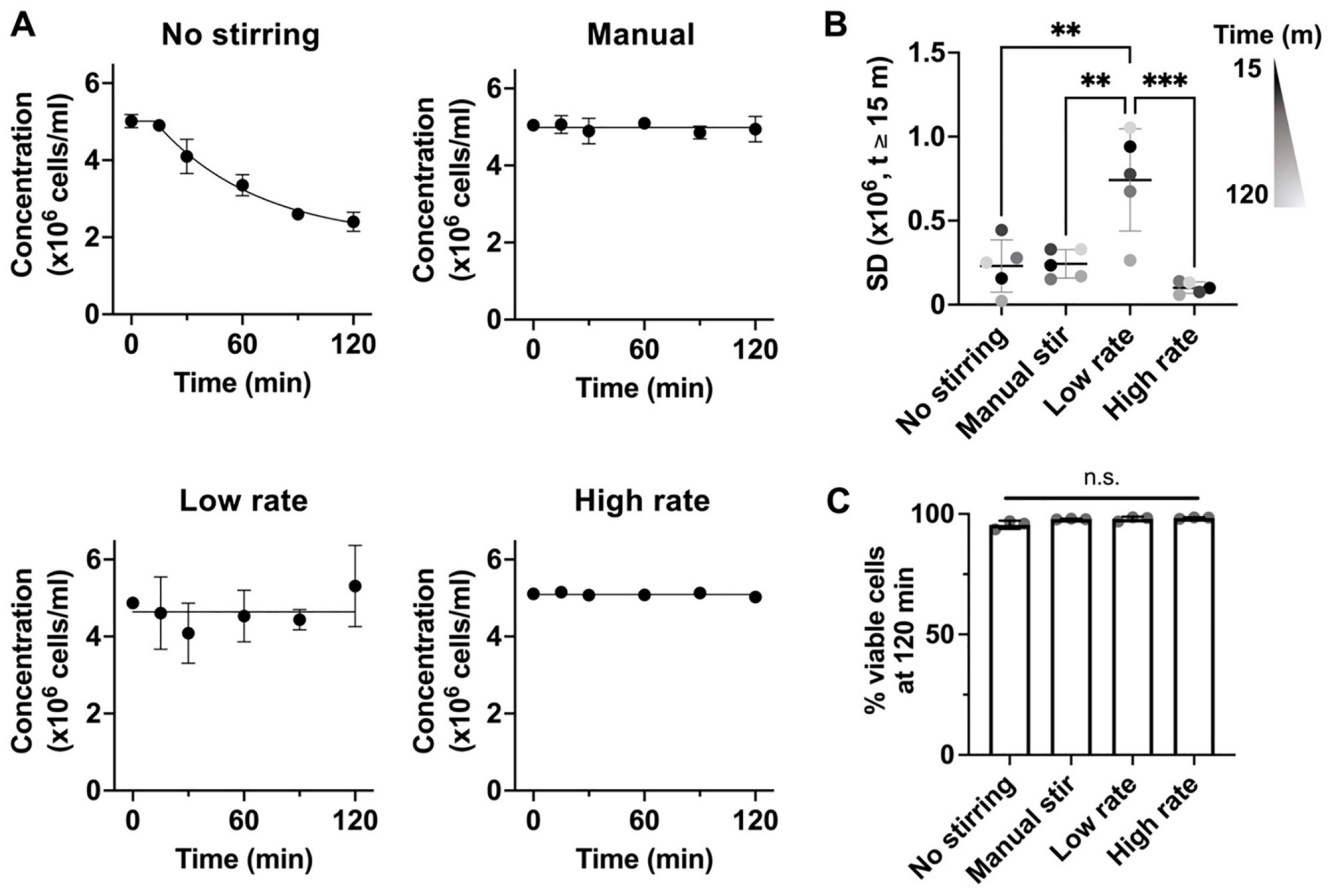
Automated stirring maintains cell concentration over time and cell viability. (A) Cell concentrations as a function of time for each agitation condition. The data were fitted to a constant followed by one-phase exponential decay curve for no stirring (t_0_=12.4 min, t_1/2_=38.7 min, plateau after decay = 1.92 million; R^2^>0.95), and a linear curve with slope = 0 for other groups. P<0.05 via two-way ANOVA. (B) Standard deviation of cell concentrations for each agitation condition. ***p<0.001, **p<0.01 via one-way ANOVA followed by Tukey’s multiple comparisons test. (C) Percentage of viable cells at 120 minutes of agitation at agitation conditions. n = 3 independent experiments, each performed in two replicates. Error bars represent standard deviation.

To determine the ability for the agitation device to maintain cell suspension at a constant concentration, MSCs were suspended in 1% w/v alginate at 5 million cells per mL, and the cell concentration was measured over time under varying agitation (Table 3). Without agitation, MSCs started to sediment within 15 min of evaluation, and the cell concentration was decreased by more than half over 120 min of evaluation (Fig 3A). In contrast, manual agitation maintained the cell concentration over time. Continuous agitation by the device also successfully maintained the cell concentration over time. However, agitation at a lower rate resulted in more variable outcomes as shown by higher standard deviation values at different time points (Fig 3B). In all of the tested conditions, cell viability remained high (>95%) at 120 min (Fig 3C). Thus, the device can be used to replace manual agitation while maintaining cell viability.

**Table 3:** Mean and standard deviation of cell concentrations at each time point and agitation condition. Simple main effects were analyzed for each time point and agitation condition. Statistical analysis was done by two-way ANOVA.

Time (min)	No agitation (cells/mL)	Manual agitation (cells/mL)	Low-rate agitation (cells/mL)	High-rate agitation (cells/mL)	P-value (agitation)
**0**	5012500 ± 171847	5050000 ± 99216	4870833 ± 80364	5108333 ± 85086	0.153
**15**	4904167 ± 156292	5066667 ± 234632	4608333 ± 941187	5150000 ± 99216	0.577
**30**	4100000 ± 444410	4891667 ± 329378	4087500 ± 777416	5075000 ± 76034	0.068
**60**	3354167 ± 277920	5100000 ± 151554	4529167 ± 674344	5079167 ± 139381	0.020
**90**	2600000 ± 21651	4858333 ± 169712	4437500 ± 264575	5129167 ± 59073	<0.001
**120**	2404167 ± 250104	4945833 ± 329852	5312500 ± 1053565	5025000 ± 132288	0.001
**P-value (Time)**	<0.001	0.740	0.461	0.726	<0.001 (interaction)

### Agitation maintains gene expression involved in mechanotransduction at a shorter timescale

**Fig 4:**
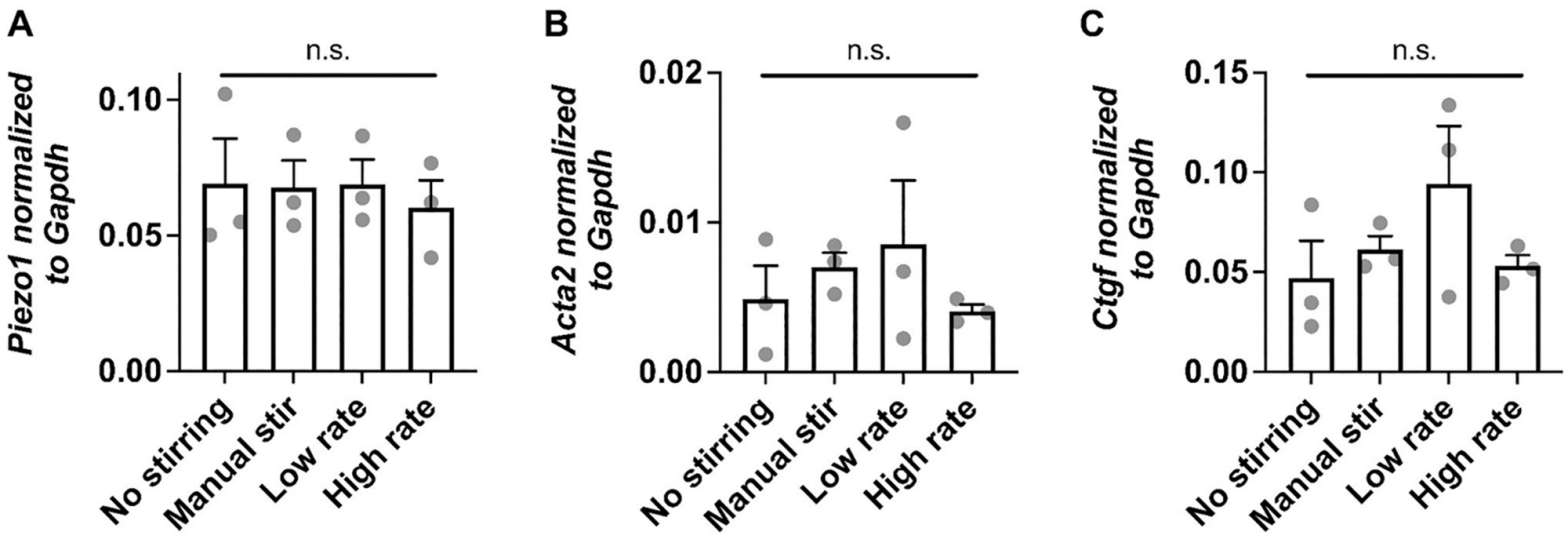
Agitation does not alter the expression of mechanosensitive genes at a shorter timescale. Gene expression analysis of (A) *Piezo1*, (B) *Acta2*, (C) *Ctgf* in MSCs from different tested conditions after 120 min. n = 3 independent experiments, each performed in three replicates. Error bars represent standard deviation.

We next assessed whether agitation impacts gene expression of MSCs in alginate suspension. We focused on testing gene expression of targets that are known to be involved in mechanotransduction, including *Piezo1*, an ion channel that is activated by force [17], actin alpha 2, smooth muscle (*Acta2*), a cytoskeletal protein that is upregulated upon mechanical activation [18], and connective tissue growth factor (*Ctgf*), one of the downstream genes of Yes-associated protein (Yap), a mechanosensitive transcription factor [19]. All the tested conditions showed the similar gene expression values after 120 min (Fig 4). Thus, agitation does not have a short-term effect on the expression of some mechanosensitive genes.

## Discussion

Success of advanced biomanufacturing based on a colloidal suspension depends on the ability to maintain the consistency of the suspension over time. For biological products, such as encapsulated cells, it is also important to ensure that their properties are not inadvertently altered during the manufacturing process. To this end, we developed a programmable, modular agitation device to maintain cell suspension during syringe pump perfusion. The agitation device is low-cost (∼$50 per unit), designed with 3D computer-aided design software, fabricated using 3D printing technologies and readily available hardware (e.g., motor, threaded rods, circuitry). Correspondence of the control settings (i.e., linear motion) with that of the actual agitation profile was established. In testing, the device can automatically maintain cell suspension without impacting cell viability, consistent with manual agitation. Since cell sedimentation under gravity without agitation depends on intrinsic cell size and density in suspension [20], our device can be used to systematically determine the optimal agitation rate for each cell type. Although our results show that the expression of the tested mechanosensitive genes is not altered after 2 h of continuous agitation, our platform can be leveraged for more detailed investigations into the impact of shear force on the gene expression kinetics over different timescales, which remains to be better defined for mechanosensitive genes [21]. For certain applications, the optimization of linear translation rate and variable time delay may be advantageous to ensure homogenous suspension with minimal energy transfer. The device can also be programmed for future studies to implement a variable time delay between each translation cycle to further minimize energy transfer to the liquid and suspended particles. Notably, use of the device has several other practical benefits over the current standard, namely, convenience, availability, and scalability for longer perfusion protocols, and more precise agitation profiles which may be advantageous in certain applications.

## Supporting information

S1 Video Automated agitation device video. This video demonstrates a closeup view of the automated agitation device as it agitates the media inside a syringe.
(MP4)

S1 Dataset (XLSX)
